# Supplementary material for: They all rock: A systematic comparison of conformational movements in LeuT-fold transporters
Source: bioRxiv. 2024 Jan 29:2024.01.24.577062. Preprint. [Version 1] doi: 10.1101/2024.01.24.577062 (PMC10862720; doi:10.1101/2024.01.24.577062)
Supplement: Supplement 1 [file media-1.pdf]

They all rock: A systematic comparison of conformational movements in LeuT-fold transporters

Jacob A. Licht[1], Samuel P. Berry[1], Michael A. Gutierrez[1,2], and Rachelle Gaudet[1,3,]*

Affiliations

[1]Department of Molecular and Cellular Biology, Harvard University, Cambridge, MA, USA

[2]Present address: Novartis Biomedical Research, Cambridge, MA, USA

[3]Lead contact

* Correspondence: (gaudet@mcb.harvard.edu)

Supplementary Note: Literature review

Existing analyses of LeuT-fold proteins have identified four main conformational differences that occur during the transport cycle (Table S1 and references within): TM10a moves toward the hash and bundle to block the outer vestibule; the bundle rotates relative to the hash to close the outer vestibule and open the inner vestibule; TM5 moves away from the inner vestibule; and TM1a swings sharply up and away from the inner vestibule, possibly allowing substrate release. Each movement has been observed in at least three different LeuT-fold families to date. However, these four conformational movements are not uniform, and they can be more pronounced in certain proteins than others and can occur in different order, relative both to each other and to substrate binding and release.

The O_{op}→O_{oc} transition involves movement of TM6a and TM10a

The O_{op}→O_{oc} transition occurs after the binding of substrate (and any co-substrate) in importers[1]. During this transition, TM6a (in AdiC and SERT) or TM10a (in AdiC, SERT, Mhp1, and in MntH for the O_{op}→I_{oc} comparison) may move into the outer vestibule and rotate toward the membrane plane (Table S1)[2-4]. For Mhp1, TM10a moves toward the outer bundle in both the O_{op}→O_{oc} and O_{oc}→I_{op} comparisons, suggesting that its movement can span multiple transitions[2-4]. In all O_{oc} structures, the binding site is blocked either by TM6a's proximity to the hash or TM10's interaction with TM1b, explaining the necessity of movement by TM6a or TM10a (Table S2).

The outward-to-inward transition involves bundle-hash movement and movement of arms

The outward-to-inward transition involves a continuation of the movements from the O_{op}→O_{oc} transition that close the outer vestibule as well as movements to open the inner vestibule. In all

LeuT-fold transporters with structures in multiple conformations, except BetP, previous analyses have noted that the bundle tilts relative to the hash, closing the outer vestibule and opening the inner vestibule (Table S1). During this movement, the angle between the bundle helices and TM4 of the hash decreases[5]. In LeuT, SERT, Mhp1, and LAT1, the entire bundle moves toward the hash in the outer vestibule and away from the hash in the inner vestibule[3,6-8]. In contrast, TM6a is the only bundle helix that has been reported to move independently during the outward-to-inward transition, in MntH[9]. In BetP, the outward-to-inward transition has been described as the outer portion of TM3 moving closer to and the inner portion of TM9 moving away from the bundle instead of the bundle moving relative to the hash[10]. Similarly, in KCC1 rocking of TM3 and TM8, as well as movement of TM4 and TM9, allow for alternating access[11]. The bundle can move either like a rigid body, like in Mhp1 where the I_{op} structure superimposes almost exactly with a model based on a screw transformation of the bundle from the O_{op} structure[12] or with independent helix movements, such as in MntH[9].

The outward-to-inward transition also involves movement of TM5 away from the inner hash to open the inner vestibule, as seen in SERT, LeuT, Mhp1, and MntH (Table S1). TM5 movement is also seen in the $I_{oc} \rightarrow I_{op}$ comparisons for SGLT and LeuT, and in $O_{op} \rightarrow I_{oc}$ for MntH (Table S1). The TM5 movement is greater in Mhp1 than in SGLT and LeuT (Table S1). The movement of TM5 away from the inner vestibule in SERT was proposed to allow Na^+ release[3].

The $I_{oc} \rightarrow I_{op}$ transition involves swinging of TM1a

Finally, the $I_{oc} \rightarrow I_{op}$ transition involves an independent swinging movement of TM1a up and away from the bundle in LeuT, SGLT, SERT, and MntH (Table S1)[3,5,6,13]. Deletion of TM1a interfered with transport in the *Deinococcus radiodurans* homolog of MntH[13] but did not completely abrogate transport in the *Staphylococcus capitis* homolog[14]. In site-directed spin

labelling experiments with LeuT, TM1a did not show as large a magnitude of movement as expected from the structural comparison of the $O_{oc} \rightarrow I_{op}$ structures, leading to the suggestion that the structural data may overestimate this movement[6].

Table S1. Conformational movements of described in literature. Unless otherwise noted, the outward to inward transition is described.

**Outer bundle helices**					
**Protein**	**TM1b**	**TM2a**	**TM6a**	**TM7b**	**Refs**
AdiC		moves toward the hash during $O_{op}\rightarrow O_{oc}$ transition	moves (more than other helices) toward the hash to occlude the binding site from the outer vestibule		2
BetP	bundle undergoes little movement throughout transport cycle				10
KCC1	bundle undergoes little movement throughout transport cycle				11
LAT1	bundle rotates 27º relative to hash				8
LeuT	moves toward TM3 and TM5 of the scaffold		moves toward TM3 and TM5 of the scaffold	moves toward TM3 and TM5 of the scaffold	6
Mhp1	bundle and hash undergo little movement during outward occlusion, but hash rotates 30º with respect to the bundle as a rigid body during outward closing				7
MntH	compared to other transporters, less movement of outer bundle helices, and bundle and hash do not move as strict rigid bodies				9
			only mobile helix among bundle helices		
SERT	rotates 22° and translates 5.1 Å towards the hash	rotates 7.3° and translates 2.8 Å towards the hash	rotates 3° and translates 1.9 Å towards the hash during the $O_{op}\rightarrow O_{oc}$ transition; rotates 5° and translates 3.4 Å towards the hash during the $O_{oc}\rightarrow I_{op}$ transition	rotates 4.8° and translates 1.0 Å towards the hash	3

**Inner bundle helices**					
**Protein**	**TM1a**	**TM2b**	**TM6b**	**TM7a**	
BetP	bundle undergoes little movement throughout transport cycle				10
	inner vestibule in I_{op} is narrower than in other transporters; negatively charged membrane lipid restricts the movement of the bundle				15
KCC1	bundle undergoes little movement throughout transport cycle				11
LAT1		hydrogen bond with TM10b is broken			8
LeuT	rotates 11º in plane of membrane from $O_{oc}\rightarrow I_{oc}$; 136º kink in O_{oc} and 125º kink in I_{op}; N-terminus of TM1a unwind from $I_{oc}\rightarrow I_{op}$		moves upon substrate binding to open inner vestibule; Y268 necessary for inward-closing movement of TM5	moves away from TM4 and intracellular loop between TM2 and TM3	6,9,16
Mhp1	hash rotates 30º with respect to the bundle as a rigid body during outward closing				7
MntH	103º kink in I_{op}	mostly stationary during transport			9,13
SERT	rotates 40° in plane of membrane, which disrupts interactions between the N terminus and TM6b that occur in outward conformations				5
SGLT	kinks outward during transition to I_{op}				5

**TM5 and TM10**			
**Protein**	**TM5**	**TM10**	**Refs**

AdiC		moves inward to obstruct the outer vestibule	2
LAT1		hydrogen bond with TM2 breaks; inhibitor blocks the rotation of TM10 in O_{op} conformation	8
LeuT	angle between TM5 and membrane plane decreases by 30.0º		5
	responsible for inward closing movement		6
	loop between TM4 and TM5 extends, so TM5 tilts away from TM1 from $O_{oc} \rightarrow I_{oc}$, which solvates Na^+ binding site		16
Mhp1	angle between TM5 and membrane plane decreases by 24.1º from $O_{oc} \rightarrow I_{op}$; rotates out and away from the cytosolic pathway	angle between TM10 and membrane plane increases in total by 36º (roughly equally during the $O_{op} \rightarrow O_{oc}$ and outward to inward steps); moves inward to obstruct the outer vestibule	5
	less flexible when outward conformation stabilized	flexibility increases when outward conformation stabilized	4
MntH	shows large movement	outer half shows large movement	9
SERT	rotates 3.2° and translates 1.3 Å toward the bundle during outward occlusion; rotates 7º from outward to inward; TM5a and TM5b translate 1.8 Å and 1.0 Å away from the binding site, respectively	rotates 2° and translates 0.9 Å towards the bundle during outward occlusion	3
SGLT	movement away from hash facilitates inward closing, but less in SGLT and LeuT than in Mhp1	predicted to move 16 Å to obstruct the outer vestibule	17

Table S2. Literature-based identification of the helices or sidechains that occlude the binding site in each occluded structure. Occlusion can be mediated by entire helices or by a single sidechain.

Conformation	Protein	Structure	What occludes binding site?	Refs
O_{oc}	AdiC	3L1L	TM6a moves, displacing the W202 sidechain 10 Å from $O_{op} \rightarrow O_{oc}$, which blocks outer vestibule[1]	2
	BetP	4AIN	W377 (TM6a) rotates by 90º to block outer vestibule[2]	10
	Mhp1	4D1B	TM10b folds over the binding site to occlude substrate[3]	18
	SERT	6DZV	Movement of TM6a toward scaffold occludes binding site[4]	3
	LeuT	2A65	Substrate is directly occluded from outer vestibule by sidechains of Y108 (TM3) and F253 (TM6a), and above those sidechains is an ion pair of R30 (TM1b) and D404 (TM10a)[5]	19
I_{oc}		6XWM	Y268 (TM6b), Q361, and D369 (both TM8) occlude the inner vestibule[6]	16
	SGLT	3DH4	M73, Y87 (both TM1a), and F424 (TM8) occlude the substrate from the inner vestibule[7]	20
	*Dr*MntH	6C3I	TM1a moves away from TM8 to solvate the binding site from the interior aqueous vestibule[8]	21

Table S3. List of available comparisons

	**Conformations**				**Comparisons**					
**Protein**	$\mathbf{O_{op}}$	$\mathbf{O_{oc}}$	$\mathbf{I_{oc}}$	$\mathbf{I_{op}}$	$\mathbf{O_{op} \rightarrow I_{op}}$	$\mathbf{O_{op} \rightarrow O_{oc}}$	$\mathbf{O_{op} \rightarrow I_{oc}}$	$\mathbf{O_{oc} \rightarrow I_{oc}}$	$\mathbf{O_{oc} \rightarrow I_{op}}$	$\mathbf{I_{oc} \rightarrow I_{op}}$
*Ec*AdiC	X	X				X				
*Cg*BetP	X	X		X	X	X			X	
*Dr*MntH	X		X	X	X		X			X
*Hs*SERT	X	X		X	X	X			X	
*Aa*LeuT	X	X	X	X	X	X	X	X	X	X
*Ml*Mhp1	X	X		X	X	X			X	
*Vp*SGLT			X	X						X
*Hs*KCC1	X			X	X					
*Hs*LAT1	X			X	X					
**Total**	**8**	**5**	**3**	**8**	**7**	**5**	**2**	**1**	**4**	**3**

Table S4. Transmembrane helix ranges determined for each protein

TM	LeuT	SERT	BetP	Mhp1	MntH	KCC1	LAT1	AdiC	SGLT
1a	13-21	85-96	139-147	30-41	46-54	121-133	53-64	13-24	54-65
1b	27-38	103-111	152-168	43-55	57-70	137-147	67-76	27-36	67-80
2	43-69	117-141	177-210	59-86	74-100	148-174	86-108	43-65	82-109
3	88-121	159-185	235-266	103-134	114-146	196-225	127-153	84-109	125-158
4	167-184	255-269	280-292	142-159	150-165	250-268	171-188	125-142	161-176
5a	191-198	279-288	301-306	161-174	175-182	272-282	192-202	146-156	187-198
5b	199-209	289-299	312-324	175-186	183-194	283-293	203-214	157-168	199-210
6a	241-254	325-337	366-373	208-220	216-227	419-428	242-251	192-201	249-264
6b	260-267	342-350	379-387	222-232	231-236	434-440	258-264	208-214	269-276
7	276-306	360-385	394-426	246-278	257-286	448-471	271-294	221-244	280-312
8	338-370	422-450	452-479	296-329	311-340	504-532	325-352	275-303	349-380
9	375-396	464-479	492-506	337-349	352-365	555-569	374-388	324-339	394-414
10a	398-407	487-495	515-524	359-369	370-383	576-583	395-402	352-359	422-432
10b	409-423	500-513	527-538	370-382	384-396	584-597	403-416	360-373	434-447

Table S5. Structures used as inputs for DALI searches

Protein	Structure (PDB ID including chain)	Hits
AdiC	5J4NA	246
ApcT	3GIAA	298
BetP	4DOJA	296
CaiT	2WSWA	297
DAT	4XPFA	297
GadC	4DJKA	298
LeuT	2Q6HA	298
Mhp1	4D1DA	272
MhsT	4US3A	271
MntH	6C3IA	296
SERT	6AWNA	238
SGLT	3DH4A	296

Table S6. Statistics of RMSDD value distributions for intrahelical and interhelical comparisons.

			K-S test statistics					
			Intrahelical vs. intrahelical		Interhelical vs. intrahelical		Interhelical vs. interhelical	
Helix	Mean RMSDD (Å)	Standard deviation (Å)	*statistic*	*p-value*	*statistic*	*p-value*	*statistic*	*p-value*
1a	0.503	0.381	0.262	0.103	0.558	6.35E-07	0.245	1.88E-14
1b	0.323	0.174	0.129	0.848	0.682	1.27E-10	0.08	0.063
2	0.351	0.195	0.171	0.539	0.667	4.23E-10	0.116	0.002
3	0.389	0.195	0.189	0.417	0.675	2.31E-10	0.096	0.014
4	0.427	0.503	0.136	0.802	0.61	2.46E-08	0.065	0.199
5a	0.447	0.367	0.168	0.565	0.507	9.92E-06	0.184	2.51E-08
5b	0.355	0.31	0.252	0.129	0.711	1.16E-11	0.102	0.007
6a	0.468	0.399	0.136	0.802	0.539	1.79E-06	0.071	0.132
6b	0.325	0.219	0.175	0.514	0.673	2.56E-10	0.114	0.002
7	0.458	0.242	0.378	0.004	0.575	2.33E-07	0.103	0.006
8	0.43	0.189	0.315	0.028	0.62	1.24E-08	0.1	0.009
9	0.333	0.259	0.199	0.352	0.68	1.51E-10	0.055	0.395
10a	0.482	0.587	0.164	0.592	0.594	7.13E-08	0.214	4.31E-11
10b	0.245	0.13	0.325	0.021	0.779	1.57E-14	0.158	2.93E-06

Table S7. Angles between predicted membrane planes of transporter structures in O_{op} and I_{op} conformations when aligning by distinct structural element

Protein	Number of LeuT-fold protomers in native oligomer	Bundle		Hash		Core	
		RMSD (Å)	θ (°)	RMSD (Å)	θ (°)	RMSD (Å)	θ (°)
LeuT	monomer[22]	3.1	19	0.7	4	3.4	7
SERT	monomer[23]	2.7	16	1.0	4	2.8	9
BetP	trimer[24]	1.2	5	1.6	9	2.1	1
Mhp1	monomer[25]	0.6	12	0.9	15	3.1	3
MntH	monomer[14]	2.2	8	0.9	5	3.0	3
KCC1	monomer[a]	0.9	11	0.7	12	2.3	5
LAT1	monomer[26]	2.1	17	1.2	11	3.3	7

[a]KCC1 can adopt a monomeric or dimeric state under native conditions[27]. Monomer was used because PPM3.0 cannot fit both protomers of the dimeric O_{op} structure (PDB: 7TTI) into a planar membrane.

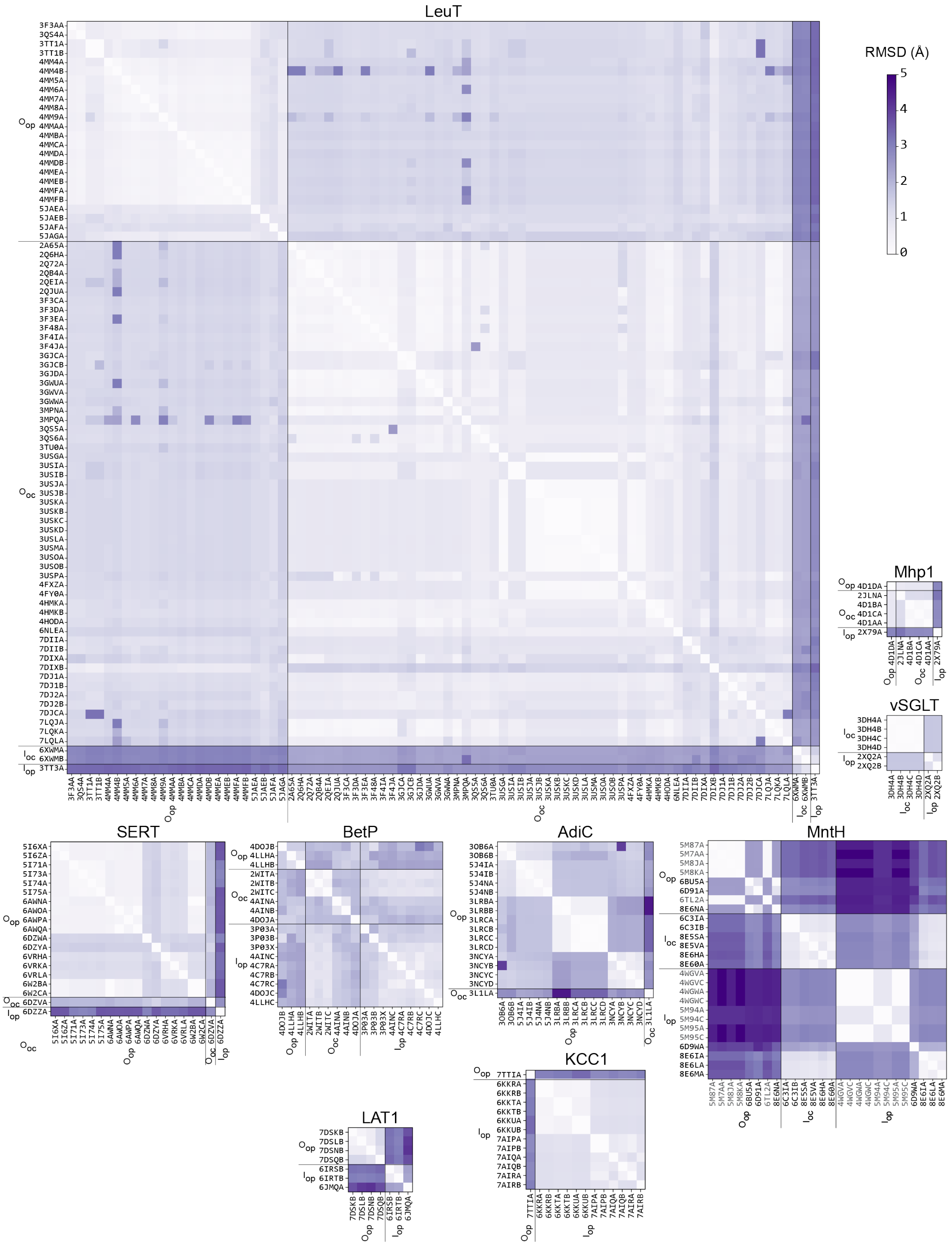

Figure S1. RMSD matrices for LeuT-fold transporters with structures in more than one conformation.

Conformations were assigned through a combination of clustering of similar structures and information gathering from the relevant publications. For KCC1, the RMSD matrices were constructed based on alignment of its transporter domain (omitting the C-terminal cytosolic domain), i.e., up to residue 655. In the case of MntH, PDB IDs in black correspond to *Deinococcus radiodurans* (Dra)MntH, and PDB IDs in grey to *Staphylococcus capitis* or *Eremococcus coleocola* MntH.

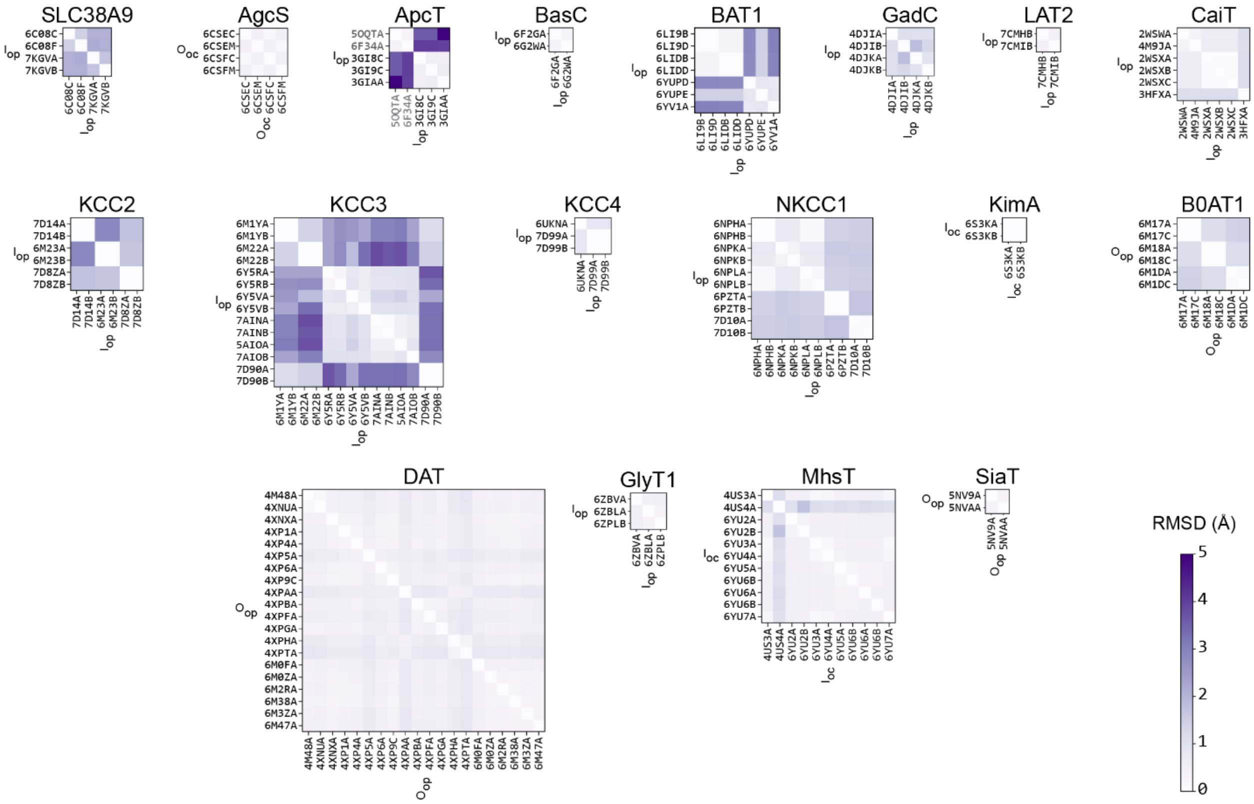

Figure S2. RMSD matrices for LeuT-fold transporters with multiple structures assigned to the same conformation.

Conformations were assigned from information gathering from the relevant publications and visual confirmation. For the CCC-family transporters, the RMSD matrices were constructed based on alignment of their transporter domains (omitting their C-terminal cytosolic domains), i.e., up to residue 660 (KCC2 and KCC4), or 675 (KCC3 and NKCC1). In the case of ApcT, PDB ID codes in grey correspond to *Geobacillus kaustophilus* ApcT, and those in black to *Methanocaldococcus jannaschii*.

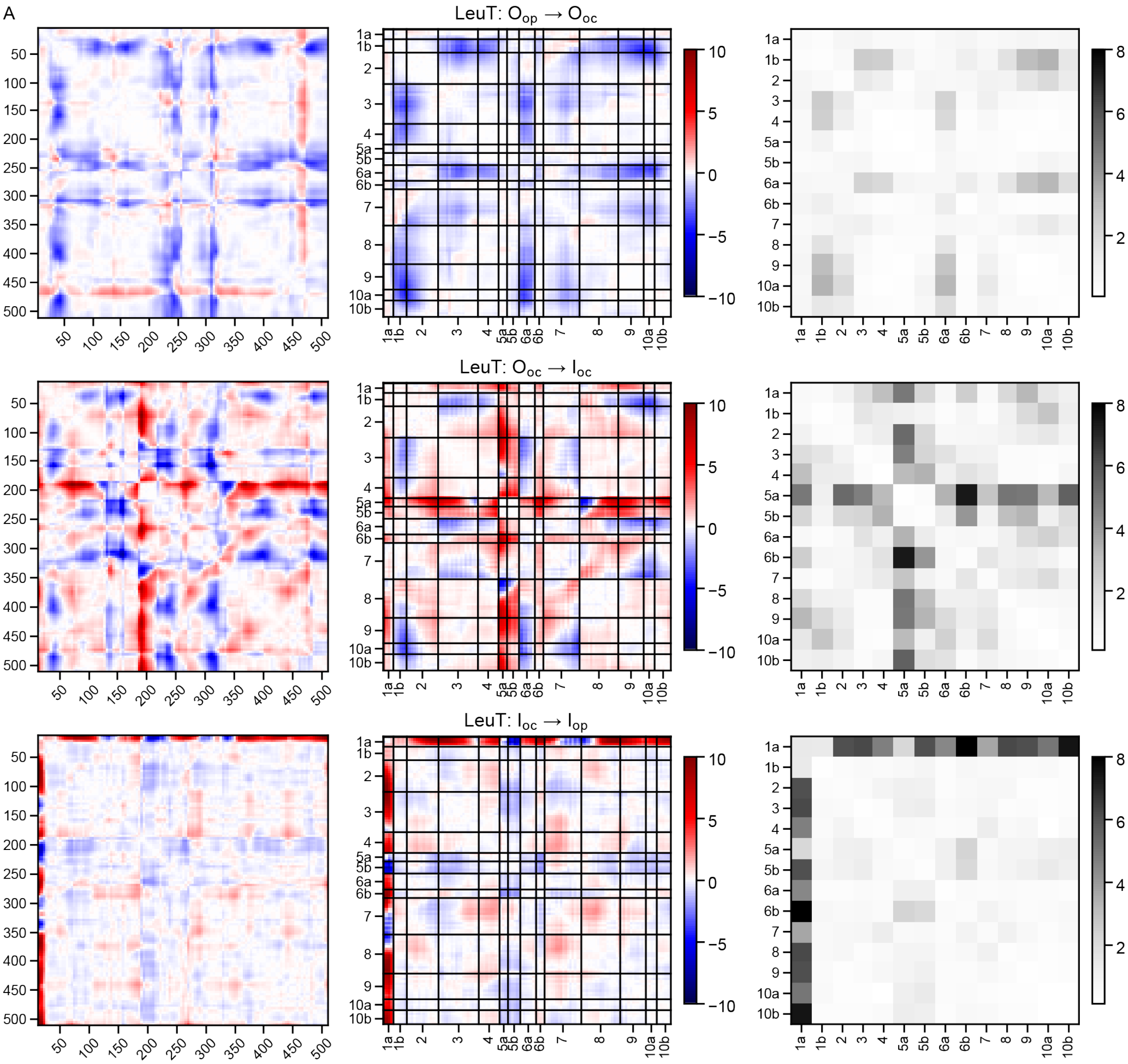

Figure S3. All DDM matrices for the 22 pairwise structural comparisons (this page and the following 6 pages).

(A) DDM matrices for LeuT one-step transitions. (B) DDM matrices for LeuT two-step and three-step transitions. (C) DDM matrices for SERT. (D) DDM matrices for BetP. (E) DDM matrices for Mhp1. (F) DDM matrices for MntH. (G) DDM matrices for KCC1. (H) DDM matrices for LAT1. (I) DDM matrices for AdiC. (J) DDM matrices for SGLT.

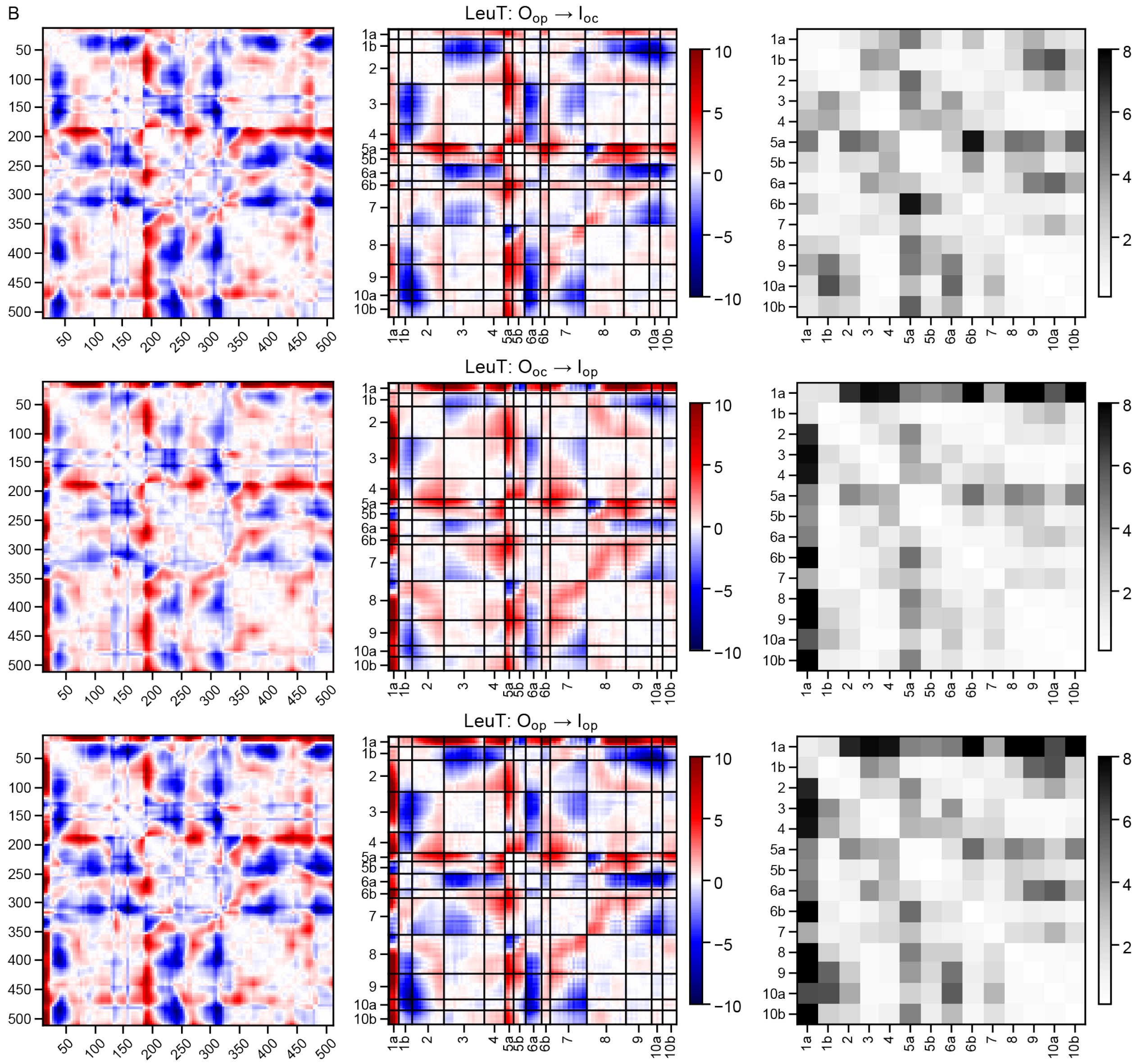
B
LeuT: $O_{op} \rightarrow I_{oc}$
LeuT: $O_{oc} \rightarrow I_{op}$
LeuT: $O_{op} \rightarrow I_{op}$

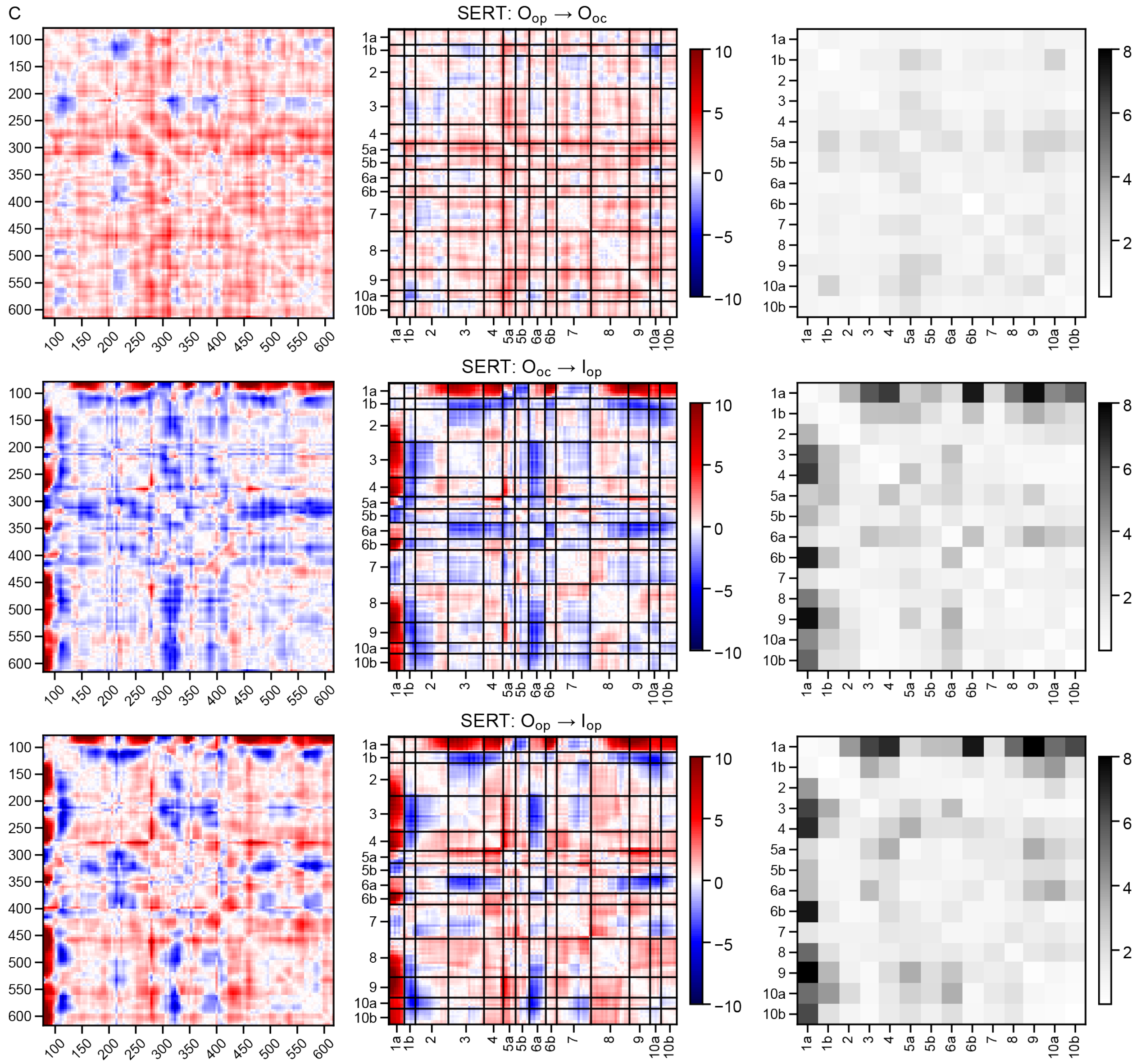
C
SERT: $O_{op} \rightarrow O_{oc}$
SERT: $O_{oc} \rightarrow I_{op}$
SERT: $O_{op} \rightarrow I_{op}$
1a
1b
2
3
4
5a
5b
6a
6b
7
8
9
10a
10b

104

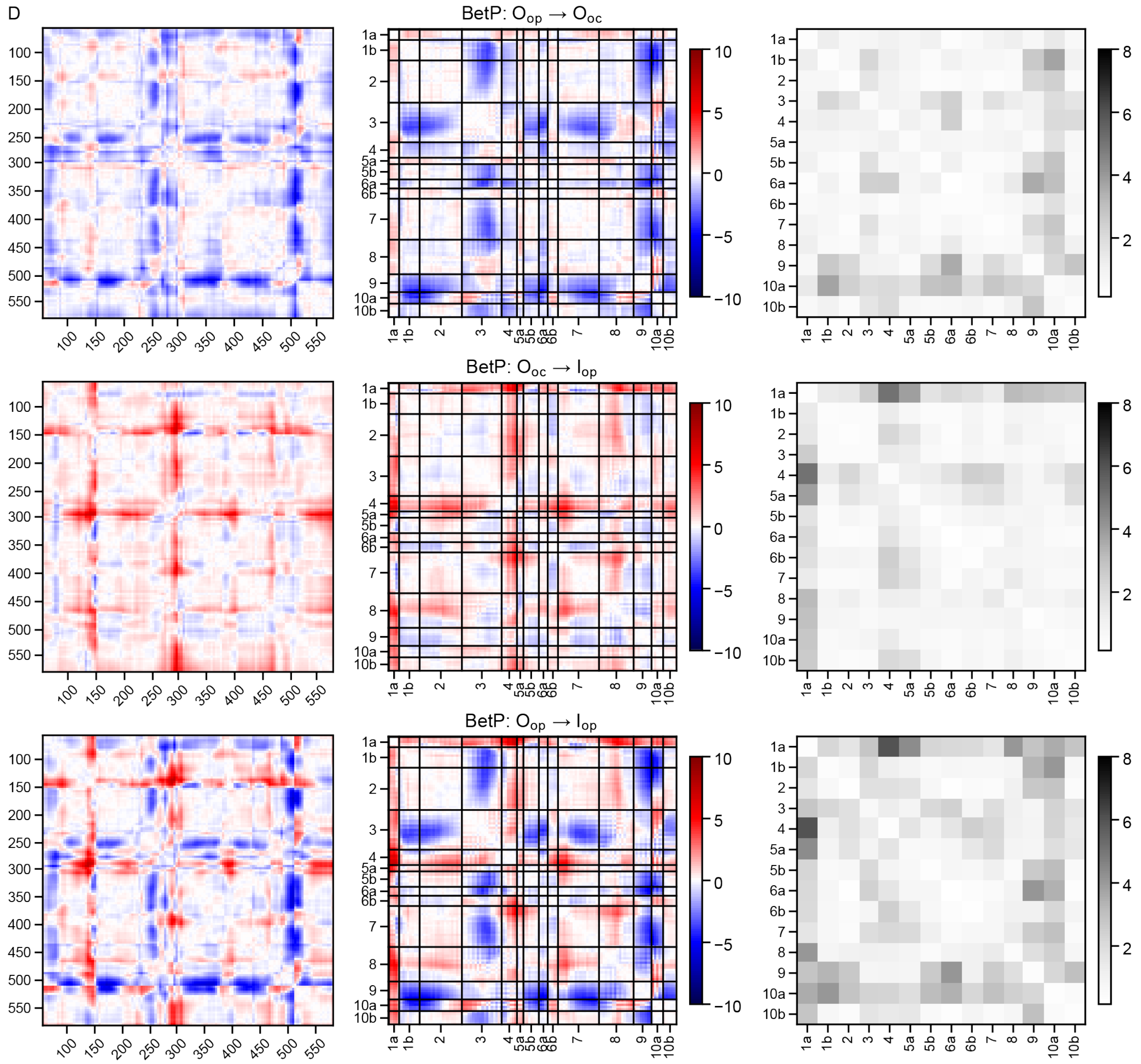
D
BetP: O_op → O_oc
BetP: O_oc → I_op
BetP: O_op → I_op

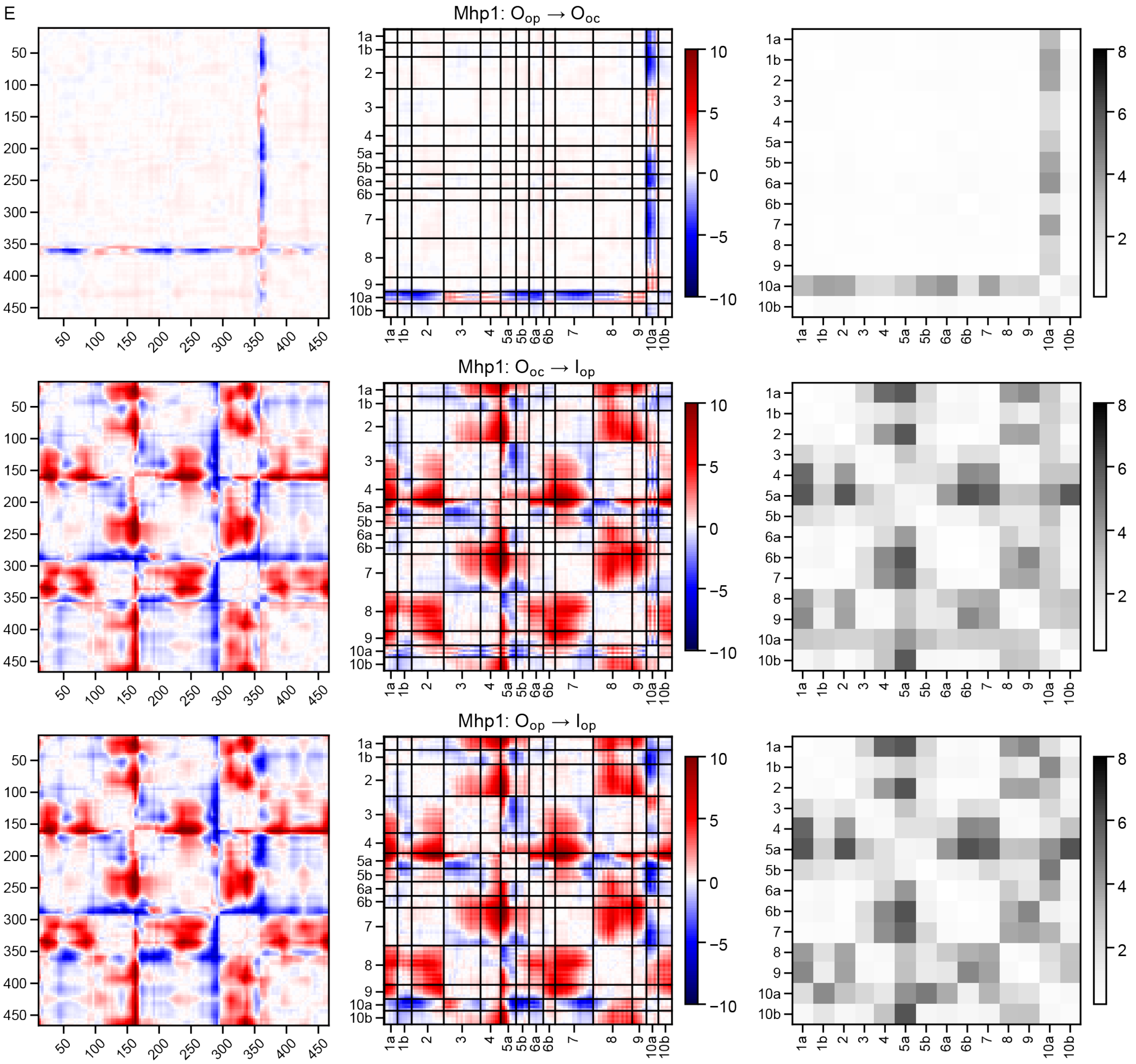
E
Mhp1: O_op → O_oc
Mhp1: O_oc → I_op
Mhp1: O_op → I_op
1a
1b
2
3
4
5a
5b
6a
6b
7
8
9
10a
10b

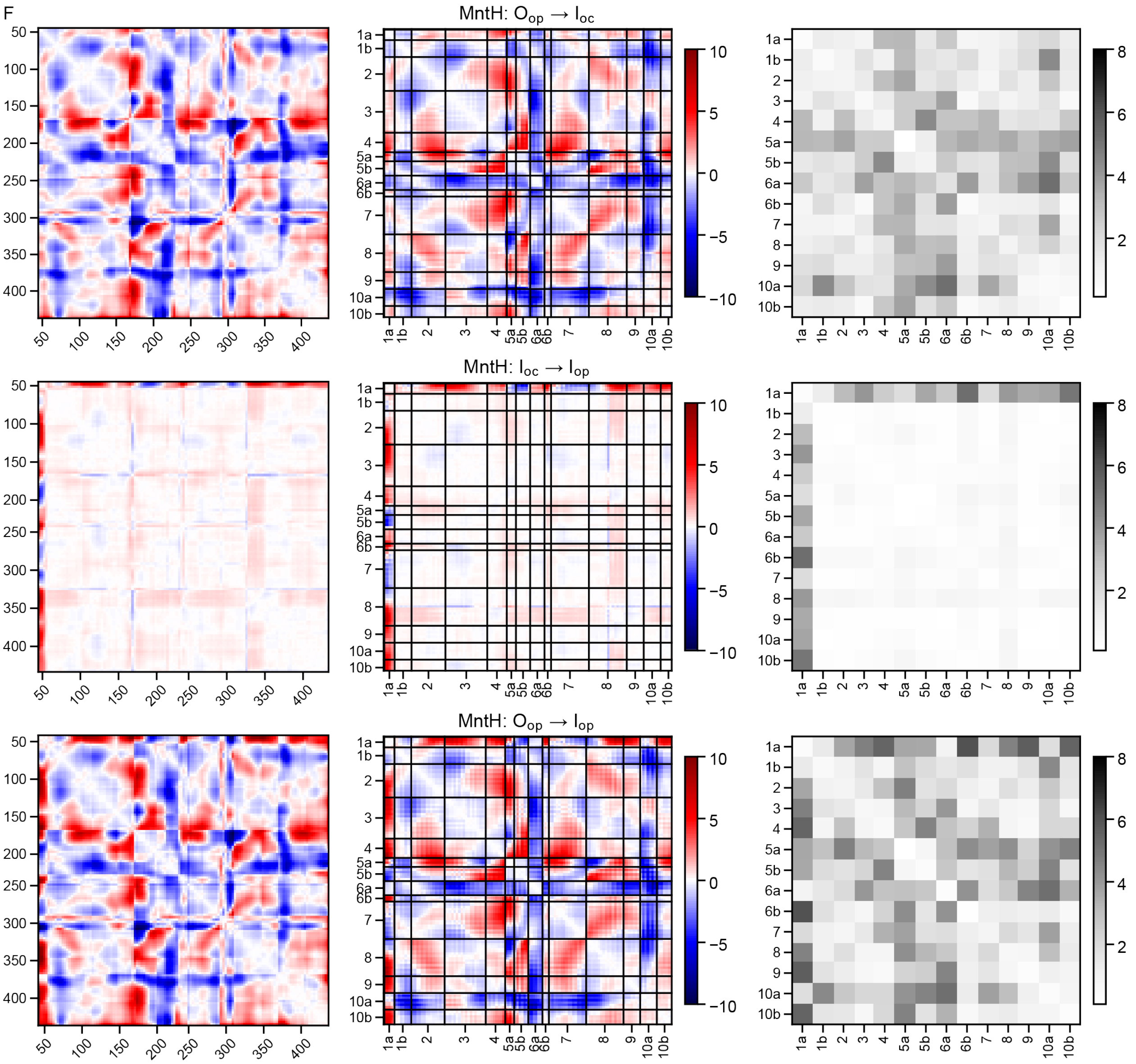
F
MntH: O_op → I_oc
MntH: I_oc → I_op
MntH: O_op → I_op

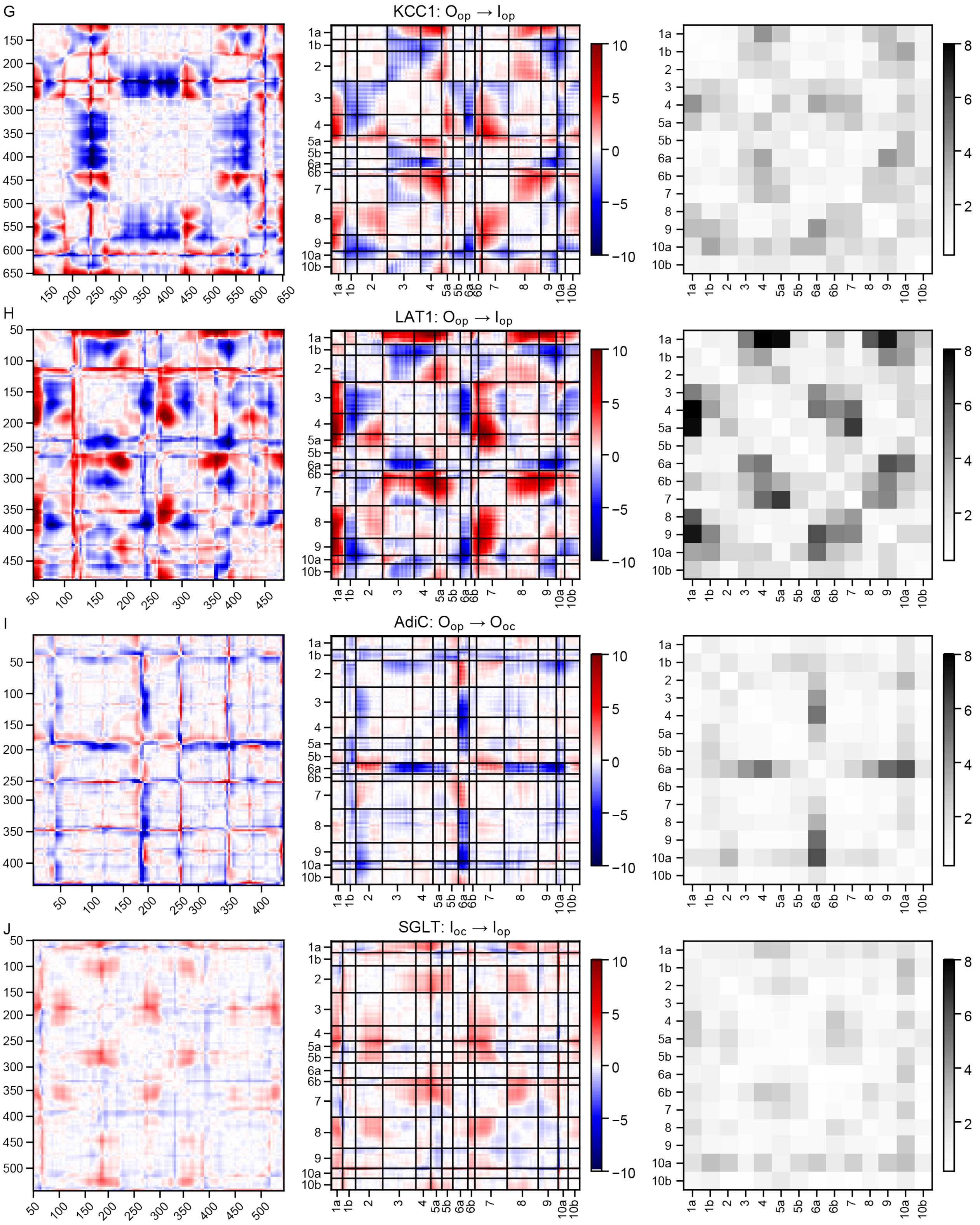
G
KCC1: Oop → Iop
H
LAT1: Oop → Iop
I
AdiC: Oop → Ooc
J
SGLT: Ioc → Iop

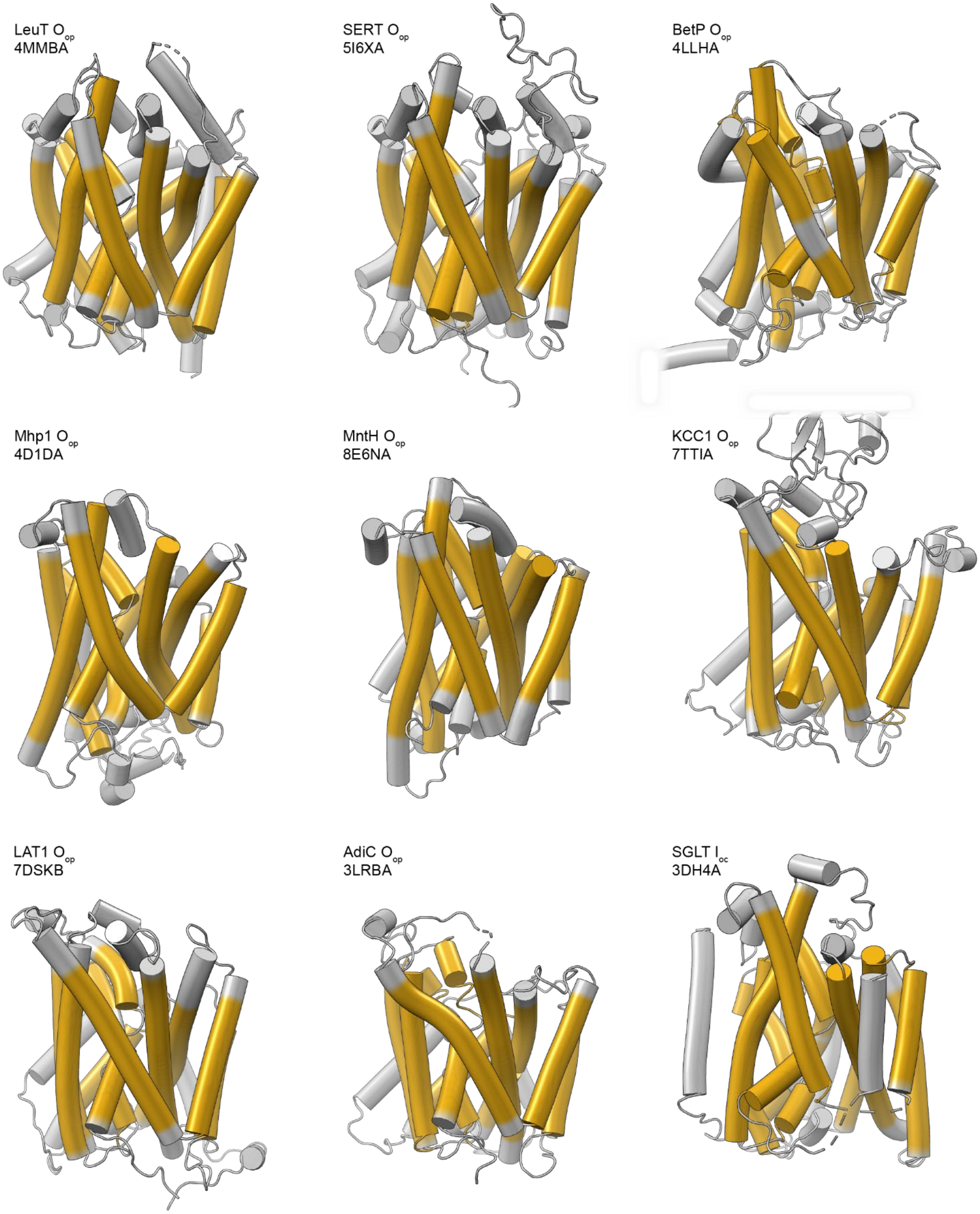

Figure S4. Illustrations of the helical residue ranges used in our analyses (yellow) for the nine proteins analyzed.

The O_{op} structures are illustrated except for SGLT, for which there is no O_{op} structure and the I_{oc} structure is illustrated instead. The C-terminal helix of BetP and extracellular domain of KCC1 are truncated in this view.

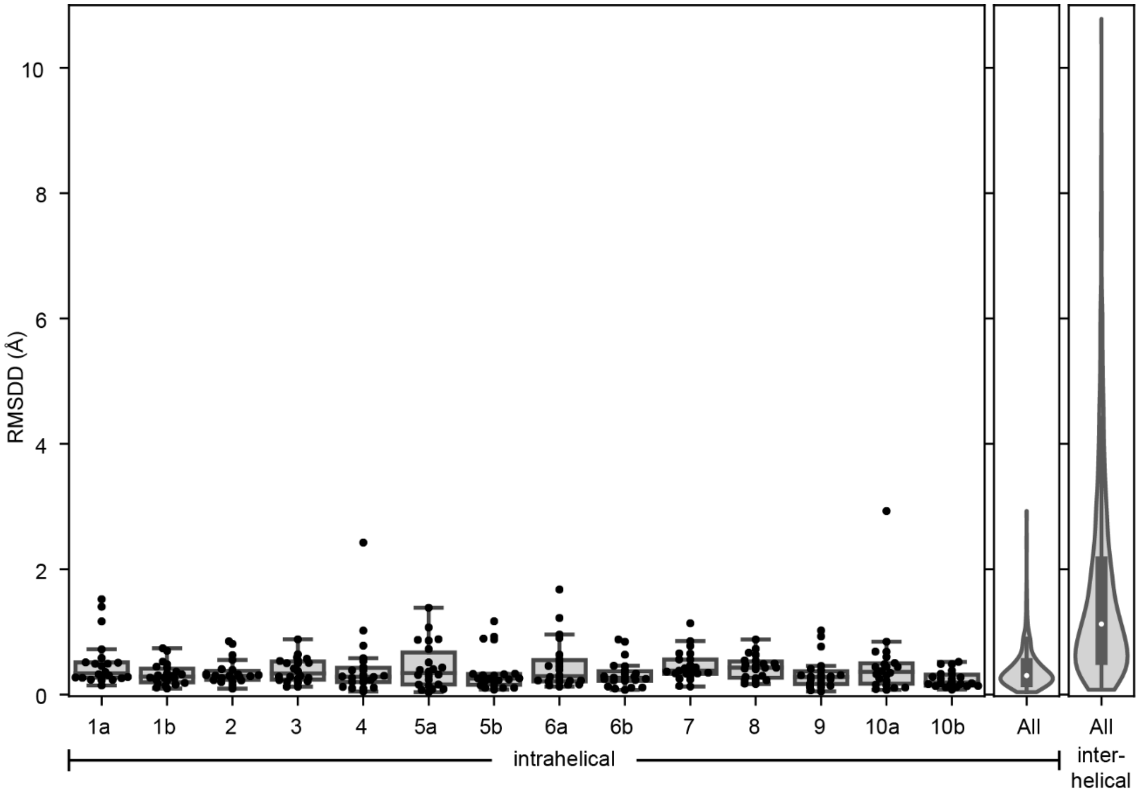

Figure S5. Intrahelix root-mean-square distance difference (RMSDD) values validate the selection of transmembrane helix ranges.

The distributions of RMSDDs for each of the 14 helices with itself (intrahelical distance differences) for all twenty-two comparisons are shown on the left, with violin plots on the right representing all intrahelical distance differences or interhelical RMSDDs. No individual helix has a distribution of intrahelical RMSDDs consistent with the interhelical RMSDDs, which would suggest that it should be subdivided. The two largest outliers are TM10a during the $I_{oc} \rightarrow I_{op}$ transition of SGLT and TM4 during the $O_{op} \rightarrow I_{op}$ transition of SERT.

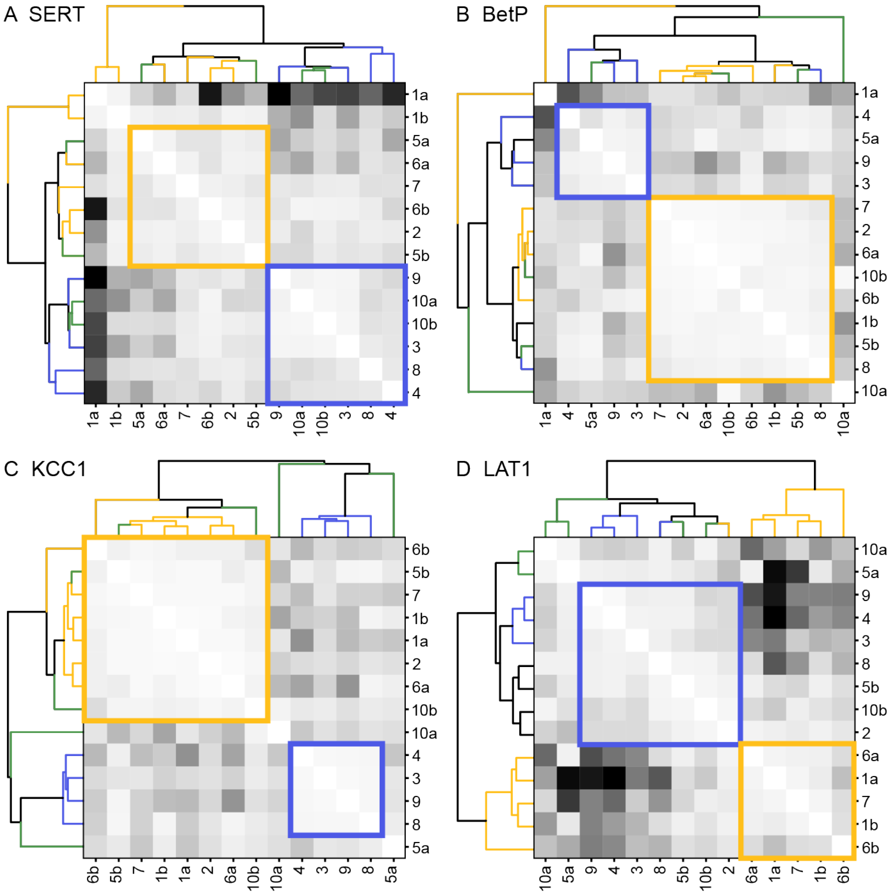

Figure S6. Clustered hb-DDMs for all available $O_{op} \rightarrow I_{op}$ comparisons show that all seven analyzed LeuT-fold transporters have a rigid hash.

This figure includes the four clustered hb-DDMs not illustrated Figure 3. The helix labels are colored blue for the hash, yellow for the bundle, and green for the arms. The rigid bodies are highlighted with colored boxes on each matrix, with blue boxes for rigid bodies containing the hash, and yellow boxes for rigid bodies containing the bundle. Only in BetP do the four hash helices (TMs 3, 4, 8, and 9) not all cluster together, with TM8 excluded from the hash cluster and clustering instead with the bundle helices.

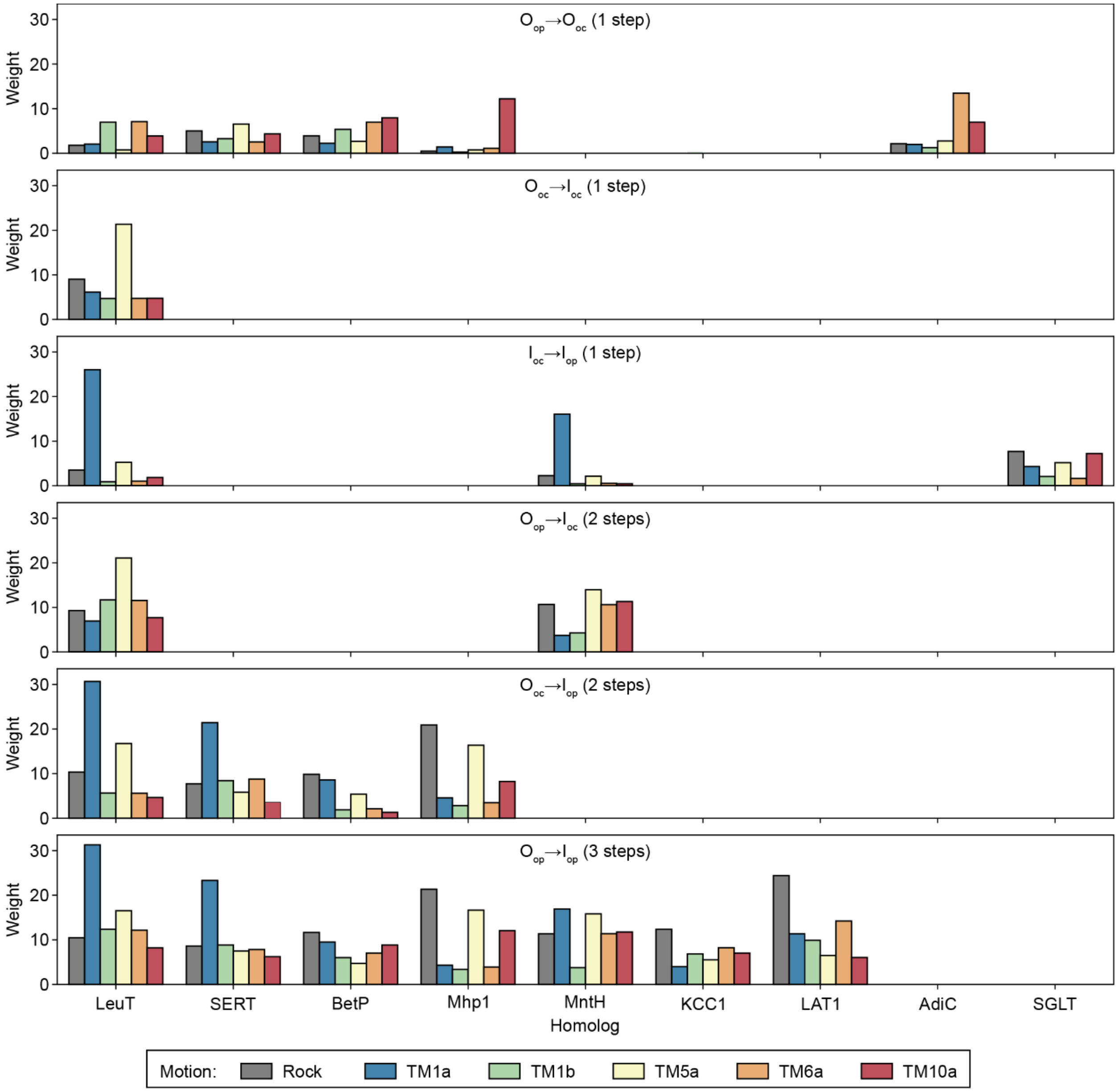

133
134 **Figure S7. Sparse PCA weights for all 22 pairwise structural comparisons.**

Supplementary Materials References

1. Forrest, L.R., Kramer, R., and Ziegler, C. (2011). The structural basis of secondary active transport mechanisms. Biochim Biophys Acta *1807*, 167-188. 10.1016/j.bbabio.2010.10.014.

2. Gao, X., Zhou, L., Jiao, X., Lu, F., Yan, C., Zeng, X., Wang, J., and Shi, Y. (2010). Mechanism of substrate recognition and transport by an amino acid antiporter. Nature *463*, 828-832. 10.1038/nature08741.

3. Coleman, J.A., Yang, D., Zhao, Z., Wen, P.C., Yoshioka, C., Tajkhorshid, E., and Gouaux, E. (2019). Serotonin transporter-ibogaine complexes illuminate mechanisms of inhibition and transport. Nature *569*, 141-145. 10.1038/s41586-019-1135-1.

4. Kazmier, K., Sharma, S., Islam, S.M., Roux, B., and McHaourab, H.S. (2014). Conformational cycle and ion-coupling mechanism of the Na^+/hydantoin transporter Mhp1. Proc Natl Acad Sci U S A *111*, 14752-14757. 10.1073/pnas.1410431111.

5. Jeschke, G. (2013). A comparative study of structures and structural transitions of secondary transporters with the LeuT fold. Eur Biophys J *42*, 181-197. 10.1007/s00249-012-0802-z.

6. Kazmier, K., Sharma, S., Quick, M., Islam, S.M., Roux, B., Weinstein, H., Javitch, J.A., and McHaourab, H.S. (2014). Conformational dynamics of ligand-dependent alternating access in LeuT. Nat Struct Mol Biol *21*, 472-479. 10.1038/nsmb.2816.

7. Shimamura, T., Weyand, S., Beckstein, O., Rutherford, N.G., Hadden, J.M., Sharples, D., Sansom, M.S., Iwata, S., Henderson, P.J., and Cameron, A.D. (2010). Molecular basis of alternating access membrane transport by the sodium-hydantoin transporter Mhp1. Science *328*, 470-473. 10.1126/science.1186303.

8. Yan, R., Li, Y., Muller, J., Zhang, Y., Singer, S., Xia, L., Zhong, X., Gertsch, J., Altmann, K.H., and Zhou, Q. (2021). Mechanism of substrate transport and inhibition of the human LAT1-4F2hc amino acid transporter. Cell Discov *7*, 16. 10.1038/s41421-021-00247-4.

9. Bozzi, A.T., Zimanyi, C.M., Nicoludis, J.M., Lee, B.K., Zhang, C.H., and Gaudet, R. (2019). Structures in multiple conformations reveal distinct transition metal and proton pathways in an Nramp transporter. eLife *8*, e41124. 10.7554/eLife.41124.

10. Perez, C., Koshy, C., Yildiz, O., and Ziegler, C. (2012). Alternating-access mechanism in conformationally asymmetric trimers of the betaine transporter BetP. Nature *490*, 126-130. 10.1038/nature11403.

11. Zhao, Y., Shen, J., Wang, Q., Ruiz Munevar, M.J., Vidossich, P., De Vivo, M., Zhou, M., and Cao, E. (2022). Structure of the human cation-chloride cotransport KCC1 in an outward-open state. Proc Natl Acad Sci U S A *119*, e2109083119. 10.1073/pnas.2109083119.

12. Forrest, L.R., and Rudnick, G. (2009). The rocking bundle: a mechanism for ion-coupled solute flux by symmetrical transporters. Physiology (Bethesda) *24*, 377-386. 10.1152/physiol.00030.2009.

13. Bozzi, A.T., Bane, L.B., Weihofen, W.A., McCabe, A.L., Singharoy, A., Chipot, C.J., Schulten, K., and Gaudet, R. (2016). Conserved methionine dictates substrate preference in Nramp-family divalent metal transporters. Proc Natl Acad Sci U S A *113*, 10310-10315. 10.1073/pnas.1607734113.

14. Ehrnstorfer, I.A., Geertsma, E.R., Pardon, E., Steyaert, J., and Dutzler, R. (2014). Crystal structure of a SLC11 (NRAMP) transporter reveals the basis for transition-metal ion transport. Nat Struct Mol Biol *21*, 990-996. 10.1038/nsmb.2904.

15. Koshy, C., Schweikhard, E.S., Gartner, R.M., Perez, C., Yildiz, O., and Ziegler, C. (2013). Structural evidence for functional lipid interactions in the betaine transporter BetP. EMBO J *32*, 3096-3105. 10.1038/emboj.2013.226.

16. Gotfryd, K., Boesen, T., Mortensen, J.S., Khelashvili, G., Quick, M., Terry, D.S., Missel, J.W., LeVine, M.V., Gourdon, P., Blanchard, S.C., et al. (2020). X-ray structure of LeuT in an inward-facing occluded conformation reveals mechanism of substrate release. Nat Commun *11*, 1005. 10.1038/s41467-020-14735-w.

17. Paz, A., Claxton, D.P., Kumar, J.P., Kazmier, K., Bisignano, P., Sharma, S., Nolte, S.A., Liwag, T.M., Nayak, V., Wright, E.M., et al. (2018). Conformational transitions of the sodium-dependent sugar transporter, vSGLT. Proc Natl Acad Sci U S A *115*, E2742-E2751. 10.1073/pnas.1718451115.

18. Simmons, K.J., Jackson, S.M., Brueckner, F., Patching, S.G., Beckstein, O., Ivanova, E., Geng, T., Weyand, S., Drew, D., Lanigan, J., et al. (2014). Molecular mechanism of ligand recognition by membrane transport protein, Mhp1. EMBO J *33*, 1831-1844. 10.15252/embj.201387557.

19. Yamashita, A., Singh, S.K., Kawate, T., Jin, Y., and Gouaux, E. (2005). Crystal structure of a bacterial homologue of Na^+/Cl^--dependent neurotransmitter transporters. Nature *437*, 215-223. 10.1038/nature03978.

20. Faham, S., Watanabe, A., Besserer, G.M., Cascio, D., Specht, A., Hirayama, B.A., Wright, E.M., and Abramson, J. (2008). The crystal structure of a sodium galactose transporter reveals mechanistic insights into Na^+/sugar symport. Science *321*, 810-814. 10.1126/science.1160406.

21. Bozzi, A.T., Bane, L.B., Zimanyi, C.M., and Gaudet, R. (2019). Unique structural features in an Nramp metal transporter impart substrate-specific proton cotransport and a kinetic bias to favor import. J Gen Physiol *151*, 1413-1429. 10.1085/jgp.201912428.

22. Gupta, K., Donlan, J.A.C., Hopper, J.T.S., Uzdavinys, P., Landreh, M., Struwe, W.B., Drew, D., Baldwin, A.J., Stansfeld, P.J., and Robinson, C.V. (2017). The role of

interfacial lipids in stabilizing membrane protein oligomers. Nature *541*, 421-424. 10.1038/nature20820.

23. Kilic, F., and Rudnick, G. (2000). Oligomerization of serotonin transporter and its functional consequences. Proc Natl Acad Sci U S A *97*, 3106-3111. 10.1073/pnas.97.7.3106.

24. Perez, C., Khafizov, K., Forrest, L.R., Kramer, R., and Ziegler, C. (2011). The role of trimerization in the osmoregulated betaine transporter BetP. EMBO Rep *12*, 804-810. 10.1038/embor.2011.102.

25. Weyand, S., Shimamura, T., Yajima, S., Suzuki, S., Mirza, O., Krusong, K., Carpenter, E.P., Rutherford, N.G., Hadden, J.M., O'Reilly, J., et al. (2008). Structure and molecular mechanism of a nucleobase-cation-symport-1 family transporter. Science *322*, 709-713. 10.1126/science.1164440.

26. Verrey, F., Closs, E.I., Wagner, C.A., Palacin, M., Endou, H., and Kanai, Y. (2004). CATs and HATs: the SLC7 family of amino acid transporters. Pflugers Arch *447*, 532-542. 10.1007/s00424-003-1086-z.

27. Casula, S., Zolotarev, A.S., Stuart-Tilley, A.K., Wilhelm, S., Shmukler, B.E., Brugnara, C., and Alper, S.L. (2009). Chemical crosslinking studies with the mouse Kcc1 K-Cl cotransporter. Blood Cells Mol Dis *42*, 233-240. 10.1016/j.bcmd.2009.01.021.
